# Frequency and impact of long wait times for family planning in public-sector healthcare facilities in Western Kenya

**DOI:** 10.1080/16549716.2022.2128305

**Published:** 2022-10-03

**Authors:** Caitlin R. Williams, Laura E. Britton, Brooke W. Bullington, Debborah Muthoki Wambua, Dickens Otieno Onyango, Katherine Tumlinson

**Affiliations:** a Department of Maternal and Child Health, Gillings School of Global Public Health, University of North Carolina at Chapel Hill, Chapel Hill, NC, USA; b Columbia University School of Nursing, New York City, NY, USA; c Department of Epidemiology, Gillings School of Global Public Health, University of North Carolina at Chapel Hill, Chapel Hill, NC, USA; d Carolina Population Center, University of North Carolina at Chapel Hill, Chapel Hill, NC, USA; e Innovations for Poverty Action-Kenya, Nairobi, Kenya; f Kisumu County Department of Health, Kisumu, Kenya; g Julius Global Health, Julius Centre for Health Sciences and Primary Care, University Medical Centre, Utrecht, Netherlands

**Keywords:** Queues, sub-Saharan Africa, contraception, women’s health, access to care

## Abstract

**Background:**

Long wait times for family planning services are a barrier to high quality care and client satisfaction. Existing literature examining family planning wait times has methodological limitations, as most studies use data collected during exit interviews, which are subject to recall, courtesy, and selection bias.

**Objective:**

We sought to employ a mixed methods approach to capture the prevalence, length, causes, and impacts of wait times for family planning services in Western Kenya.

**Methods:**

We used mystery clients, focus groups, key informant interviews, and journey mapping workshops to measure and describe family planning wait times. Fifteen mystery clients visited 60 public-sector facilities to quantitatively capture wait times. We conducted eight focus group discussions with 55 current or former family planning clients and 19 key informant interviews to understand facility-level barriers to family planning and feasible solutions. Finally, we visualized the process of seeking and providing family planning with journey mapping workshops with nine clients and 12 providers.

**Results:**

Mystery clients waited, on average, 74 minutes to be seen for family planning services. In focus group discussions and key informant interviews, three themes emerged: the nature of wait times, the impact of wait times, and how to address wait times. Clients characterized long wait times as a barrier to achieving their reproductive desires. Key informants perceived provider shortages to cause long wait times, which reduced quality of family planning services. Both providers and family planning clients suggested increasing staffing or offering specialization to decrease wait times and increase quality of care.

**Conclusion:**

Our mixed methods approach revealed that wait times for family planning services were common, could be extensive, and were viewed as a barrier to high quality of care by clients, providers, and key informants. Across the board, participants felt that addressing workforce shortages would enhance service delivery and thus promote reproductive autonomy among women in Kenya.

## Introduction

In Kenya, contraceptive prevalence has increased substantially over the last two decades [1]. Yet early and closely-spaced pregnancies remain common and nearly a fifth of married women who do not desire pregnancy are not using a modern contraceptive method [2]. Facility-level barriers can impede Kenyan women’s reproductive autonomy [3]. Long wait times are one such barrier, affecting quality of care and client satisfaction with family planning services [4].

The importance of addressing long wait times has been recognized in the context of other sexual, reproductive, maternal, newborn, child, and adolescent health concerns. Ensuring timely care has long been a program objective of maternal mortality reduction efforts [5,6]. Quality improvement projects targeting wait times exist for HIV treatment clinics [7], primary health care centers [8], and labor and delivery services [9]. Yet there is less attention to reducing wait times for family planning.

Research suggests that timely access to family planning is critical. In Kenya, data from the Kenya Service Provision Assessment estimated women in public facilities waited over an hour to receive services and long wait times were significantly associated with client dissatisfaction with family planning services [10,11]. Long wait times may also contribute to contraceptive discontinuation and non-use [12]. In addition, long wait times pose a documented barrier to successfully integrating family planning with other maternal and child health services [13,14], as well as ensuring family planning services meet the needs of vulnerable populations such as adolescents [15] and sex workers [16,17]. Finally, long wait times for family planning are pervasive, reported over a wide range of settings for several decades [18–21].

Although long wait times have been identified as a meaningful barrier to care, there are methodological limitations to existing research. Structured interviews with exiting clients currently provide the primary source of quantitative data measuring the frequency and extent of wait times [22]. In these facility-based interviews, family planning clients are asked both how long they waited to access services and whether the wait time constituted a major problem, a minor problem, or no problem at all. This format renders data vulnerable to recall bias, courtesy bias, and selection bias, particularly if those most sensitive to wait times are unable to remain at the facility to participate in an exit interview. Additionally, provider and health system perspectives on the nature and causes of wait times are limited, potentially constraining the development of interventions to address this long-standing problem.

Given these limitations, we lack precise and valid estimates of the amount of time women wait to access contraceptive care in public-sector facilities as well as important contextual information. Our study seeks to address this research gap and overcome existing limitations of data on wait times for family planning research through a mixed-methods investigation. Employing a combination of mystery clients, focus groups, key informants, and journey maps, we examine the extent (prevalence, length of wait, comparison of wait time and consultation time), perceived causes, and impact of wait times, as well as potential interventions that might be leveraged to reduce wait times in Western Kenya.

## Methods

This is a descriptive, cross-sectional study which employed a convergent mixed methods design with triangulation across data from mystery clients, focus groups, and key informants and data confirmation and corroboration using data from journey mapping workshops. Our analysis of family planning wait time is nested in a larger mixed-methods study aimed at identifying barriers to high-quality, patient-centered contraceptive care in Western Kenya. Below we describe a variety of data collection methods used to measure and describe family planning wait times. Women seeking family planning face a variety of barriers to contraceptive care and our parent study was specifically designed to identify those challenges that arise upon arrival at a healthcare facility. Our approach, therefore, is underpinned by Andersen’s Behavioral Model of Health Service Use, with a focus on health system factors [23]. This framework recognizes that the health behaviors of reproductive age women are influenced by the larger health system which may discourage or enable access to care. Using this model, we seek to identify the facility-level factors that influence contraceptive access. The duration of time spent waiting to access contraceptive care is one such factor.

### Mystery client methodology

Quantitative data measuring the length of time that women wait to receive services and the amount of time providers spend on contraceptive counseling were collected by mystery clients. In the mystery client approach, a data collector visits a facility under the guise of being a patient seeking services. In the process of receiving services, the mystery client observes the healthcare provider; data on the services received is recorded and reported by the mystery client shortly after leaving the facility. Mystery clients are ideal for measuring behaviors that providers might obscure from observation when data collection teams are present [24].

In this study, a team of 15 mystery clients visited a random sample of 60 public-sector facilities located in five of the ten counties comprising the Western and Nyanza regions of Kenya. In selecting the 60 facilities, we first stratified by county to allow for an even distribution of 12 public facilities in each county, and then by public facility type: dispensary (the smallest facility type); health center (mid-size); and sub-county or county hospital (largest type). Mystery clients were selected based on their fluency in the local language and their possession of strong recall ability. Mystery clients presented as new family planning clients. Mystery clients ranged in sociodemographic characteristics; they were aged 21–37, had zero to two children, and seven were married. Each facility was visited once by three different mystery clients, for a total of 180 mystery client visits. All mystery clients arrived at the healthcare facility at or before 8:30 am – the standard opening time – to ensure comparability of wait time across all visits. Mystery clients discreetly noted the time providers arrived at the facility, the time the provider approached them for services, and the time they finished meeting with the provider. All mystery clients recorded their observations on an electronic questionnaire within 15–30 minutes of their visit. Data on wait times are descriptive counts of the total minutes spent waiting for services and the total minutes spent with the healthcare provider. All descriptive analyses were performed using Stata 14.

#### Qualitative methodology

We conducted eight focus group discussions (FGDs) of six to eight participants each with 55 current and former family planning clients, aged 18 to 46, residing in Western Kenya. Discussions were led by trained female moderators implementing a semi-structured questionnaire designed to explore facility-level barriers to family planning.

We also conducted 19 key informant interviews (KIIs). KIIs were selected via a snowball sampling technique that began with the Head of Reproductive Health in select counties. Our KII sample includes senior staff from public and private-sector healthcare facilities and non-governmental public health organizations, as well as senior government officials tasked with planning, coordinating and supervising the implementation of reproductive health services in the county/sub-county. All KIIs were conducted by a trained enumerator using a semi-structured questionnaire designed to explore feasible and promising solutions to facility-level barriers.

Finally, we synthesized data from the parent study into two journey maps [25]: a client journey map and a provider journey map. Journey maps provide a visual representation of the process of seeking or providing family planning services. We vetted the journey maps using client and provider journey mapping workshops (CJMW and PJMW respectively). Two members of the research team (one American and one Kenyan) used a qualitative description approach to conduct conventional content analysis of FGD, KII, CJMW, and PJMW qualitative transcripts. They developed a codebook with definitions and managed data with Nvivo 11.0 (QSR International).

Additional details on the facility sampling procedure, mystery client characteristics, and the methodology for each of the study components described above are available in a previous publication [26]. Ethical approval for the study protocol was provided by the University of North Carolina at Chapel Hill and the Kenya Medical Research Institute (KEMRI).

## Results

Below we present findings from mystery clients and multiple qualitative study components. The mystery client data demonstrate the average wait time and counseling time experienced by mystery clients seeking contraceptive access. As presented below, the three themes related to wait times which emerged from our qualitative corpus were the nature of long wait times, the impact of long wait times, and potential approaches for addressing the wait times for contraceptive care.

### Participant characteristics

We conducted 19 KIIs, each of which lasted 55 minutes on average, with one private sector facility director, three senior health providers, seven senior government officials, eight private sector/NGO high-level staff. We conducted eight FGDs with 55 women, one CJMW with nine women, and one PJMW with 12 women. We conducted 180 mystery client visits at 60 facilities in five counties. Participants have been described in more detail elsewhere [26,27].

### Amount of wait time

On average, mystery clients waited 74 minutes to be seen by a provider, with wait times ranging from two minutes to more than four hours (Table 1). While about one in six mystery clients waited less than 30 minutes, approximately equal numbers waited more than two hours before being seen by a provider. Just over half (58%) of mystery clients waited an hour or more to be seen. Providers spent, on average, 11 minutes with mystery clients during contraceptive counseling, with the time of counseling ranging from one to 64 minutes. One in five mystery clients were counseled by a provider for 15 to 29 minutes. Equal numbers were counseled for less than five minutes. We did not find significant or meaningful associations between mystery client age, parity, or marital status and wait time. There was also no discernable pattern of wait time regarding facility type (clinic, dispensary, or hospital) or provider gender or cadre (data not shown).

**Table 1.** Time spent waiting to be seen and time spent with the healthcare provider among 180* mystery clients to 60** public-sector facilities in Western Kenya, 2018–2019.

	N	%
Time waiting to be seen		
Less than 15 minutes	20	11%
15–29 minutes	11	6%
30–44 minutes	23	13%
45–59 minutes	18	10%
1–2 hours	80	44%
More than 2 hours	25	14%
Missing***	3	2%
Average wait time (range)	74 minutes (2–256)	
Time spent with provider		
Less than 5 minutes	36	20%
5 to 14 minutes	96	53%
15 to 29 minutes	38	21%
More than 30 minutes	7	4%
Missing***	3	2%
Average time with provider (range)	11 minutes (1–64)	

* All mystery clients presented as new family planning clients.

** Each of the 60 facilities received visits from three different mystery clients (each facility was visited three times).

*** Reason for missing data: Three MCs were turned away at registration because all family planning methods were out of stock.

### Theme 1: nature of long wait times

In focus group discussions, women affirmed that long wait times in queues were perceived as common. ‘Sometimes you can really spend a long time at the hospital!’ (FGD participant, current family planning user, urban Kisumu county). Women were particularly frustrated when they waited a long time to be told that they could not obtain their desired method because it was stocked out or no trained provider was available to insert it.

In the provider journey mapping workshops, public sector providers confirmed that they viewed long wait times as common. They often felt that patients misunderstood the many responsibilities they manage, including administrative tasks and caring for other patients.

It makes you feel all of that – frustrated and burned out – and the client thinks you are being slow or maybe the clients feel that you are having preference to some clients, in fact not knowing that there is a client who wanted family planning and tested pregnancy test positive and the client starts crying. One day I had antenatal clinic, and the clients queueing started quarreling that why do I take so long with a patient and there is a lot to be done so I just asked them, how would you feel when you get in and before you sit down I tell you to go? (Provider journey map workshop participant)

Women believed that they waited because they were deemed lower status than patients seeking immunizations, antenatal, or pediatric care. ‘Doctors do ignore women so much. And then they don’t consider women who have gone for family planning services like someone important who has come for help’ (FGD participant, current family planning user, rural Busia county). Public sector providers in the provider journey mapping workshop specified that family planning patients would wait when other patients had more urgent clinical needs, which they characterized as ‘multitasking – you find that there is a client with an emergency and family planning clients, so you’ll opt to help in the emergency’ (Provider journey map workshop participant).

Women also believed that the personal characteristics of the patients affected providers’ willingness to make them wait:

Maybe you have just bathed well but have not applied oil. The provider looks at the outlook and that is how they judge you … You can go, then the provider tells you to enter. After entering, you explain your problem. When you ask question, he asks another client to come in and tells you to wait outside for a while. He is not bothering assisting you and you will wait until you decide to go home (Client journey map workshop participant, urban Kisumu county).

Women felt that family, friends, or neighbors of the providers received preferential treatment and were seen before other women in the queue: ‘They came and picked people from the line, the people they were familiar with. The others who remained – we were there up to one in the afternoon.’ (FGD participant, current family planning user, rural Bungoma county)

Key informants identified provider shortages as the cause of long wait times. Some participants made the distinction between shortages of providers in general and shortages of providers with specific family planning competency. Key informants attributed provider shortages to budget constraints that limited hiring and training staff. They also suggested that retired or deceased providers are not replaced. Key informants described how working conditions exhausted providers: ‘Shortage of staff also leads to burnout when a health worker is now overworked’ (Senior government official). Negative treatment of patients was described as a downstream consequence of provider shortages and burnout. ‘We can hire more staff, we can even, you know, improve the process workflows to avoid stressful situations and workloads that make people into being abusive’ (Private sector/NGO high-level staff).

### Theme 2: impact of long wait times

Long wait times could result in women not obtaining desired family planning and having unintended pregnancies. Women could respond to queues by waiting until they were seen; leaving and returning to that facility on another day; leaving to patronize other facilities instead (including private facilities, if they could afford it); or leaving and never obtaining family planning. ‘Walking, just getting there, and sitting there is what made me stop using [contraception]’ (FGD participant, discontinued family planning user, urban Bungoma county).

Women described denial of services if they did not arrive early enough in the day and being blamed for non-adherence to their family planning due dates at subsequent visits: ‘They sometimes used to say I am late, at times is true I am late. When you arrive, and maybe you are late, they say that they will only attend to those who came early and that you come another day. When you go back, you may find another person who again asks you why you did not go on that day’ (FGD participant, discontinued family planning user, urban Bungoma county). Long wait times also affected women economically, if the time they spent waiting was time in which they were not earning an income: ‘I sell fish … I leave my house at eight and return at four. You know there is no business done for that day.’ (FGD participant, current family planning user, urban Kisumu county)

Women found long wait times particularly challenging when they lacked support from their partners and were trying to obtain family planning covertly: ‘For me, my husband does not support family planning. So, as you sneak you also lack the time and upon reaching the facility you find very long queues and how you would talk to the service providers to serve you because you sneaked, and you are running out of time … That can’t happen’ (Client journey map workshop participant, urban Kisumu county).

Key informants also affirmed that long wait times negatively impacted the quality of family planning service delivery by pressuring providers to serve patients quickly. They specified that in a short visit, providers may not give adequate counseling. Public sector providers in the provider journey map workshops confirmed: ‘The long queues make you not to give high quality family planning service.’

The long wait time and low quality of care in the short visits can generate negative attitudes in both the patient and the provider:

Most of them, they don’t have time so whatever they give, the package they give that pertains to family planning is … a bit limited. You will get that at the particular time, that negative attitudes comes because me, I have come on duty as a service provider then you get that I have a line of around sixty clients who need to be serviced and because you are alone. You need to serve those clients, so you get that attitude will develop – the negative ones because of the workload, because there is no motivation … . Now to the client … They get negative attitude because of the congestion and the workload (Public sector senior healthcare provider).

Time pressure can also diminish providers’ willingness to insert long-acting reversible contraceptives (LARCs), resulting in women not obtaining their desired family planning method and being counseled to use something else:

Giving an IUCD is a long procedure. I need to do many things and maybe I am alone … The queue is very long … So sometimes you are like, ahhh, I cannot insert the IUCD, the queue is very long and I will take a lot of time insert this because you need to do the counseling, there are the examinations you need to do for you determine whether client suits, is fit to be given the IUCD … . So maybe let me give depo … ‘Mum, let me give you depo for today, next time if you come I will give you the IUCD.’ So sometimes it is, we don’t give them what they desire, we give them what we feel is for good them – which is wrong. (Senior government official)

Key informants observed that women with financial resources would often go to the private sector to avoid wait times. Two concerns were expressed about this: first, they worried family planning care was not documented by current data capture for health systems at pharmacies or drug shops, and second, most Ministry of Health clinical trainings and updates were directed towards the public sector, so providers may not have up to date skills and knowledge about family planning.

#### Theme 3: addressing long wait times

In focus groups, women had two general recommendations to reduce long wait times: increase staffing and offer specialization. ‘According to me, the number of doctors should be increased at the hospital so everybody should be attended to’ (FGD participant, current family planning user, rural Busia county). Across the data collection modes, many participants identified staff shortages as the cause: in the words of one key informant, ‘Why the queue? The workload – because providers are very few’ (Private sector/NGO high-level staff).

Women also specified that dedicating providers and hours to family planning services would reduce wait times and improve quality of care: ‘Let them establish a department that is solely responsible for the family planning … That will make it easy for us to access family planning’ (FGD participant, current family planning user, urban Kisumu county). Though not framed as a solution to the long wait times, specialization was viewed favorably: ‘You know when someone handles a specific thing it tends to be done perfectly. Yes! Then one – the issue of staffs, as a country or as a ministry, we should encourage specialization’ (Private sector/NGO high-level staff). Another suggested that specialization would encourage providers to work in the type of care delivery for which they have a passion.

Women in focus groups, key informants, and public sector providers in the provider journey map workshops indicated that addressing staff shortages was a priority. ‘If the nurses can be increased in number, family planning can be okay’ (FGD participant, current family planning user, rural Bungoma county). Fixing provider shortages was characterized as the government’s responsibility. Barriers included government unwillingness to expand the workforce, inadequate budget, and the restructuring that occurred after devolution. Public sector providers concurred that ‘staff shortages – there is nothing we can do about it, but the government could ensure we have enough staffs’ (Provider journey mapping workshop participant).

## Discussion

Our mixed-methods study sought to characterize wait times for family planning services in Western Kenya and describe the impact of long wait times on clients and providers. We found that long wait times were common, could be extensive, and were viewed unequivocally as a barrier to care. Despite this, we also found significant variability in the social meaning of wait times, and a wide constellation of subsequent impacts. Across the board, participants felt workforce shortages need to be addressed.

In our sample of 60 public-sector facilities in Western Kenya, mystery clients waited, on average, over an hour to be seen, with some waiting up to four hours. Mystery clients were typically the first in the queue; a family planning client who arrives later might wait longer to be seen, potentially making our wait times underestimates of typical wait times. The average wait time of 74 minutes is similar to that observed at Kenyan public-sector facilities in prior studies [10,11], suggesting that providers do not start attending family planning clients until 9:30–10:00 am, though facilities open at 8:30am. This may be because providers juggle providing multiple types of services at once, as previously documented in other settings [28–30]. Clients attempting to obtain contraception covertly may be particularly sensitive to long wait times [31].

Family planning clients in our study believed that they had to wait longer because providers viewed them as low status patients. Thus, in addition to posing a logistical barrier to care [32], our results suggest long wait times undermine the development of positive client-provider relationships. Providers and key informants highlighted the role of patient care activities, such as charting or providing emotional support for distressed patients, in contributing to wait times. This suggests that while providers are cognizant of wait times, they view them as an unfortunate consequence of providing quality care despite inadequate staffing and heavy workloads. Given that we found no association with patient or provider characteristics, long wait times may be a symptom of structural challenges with family planning service provision rather than solely a manifestation of interpersonal discrimination.

In our study, long wait times were linked to reduced quality of care, with rushed appointments contributing to insufficient counseling, reduced willingness to insert LARCs, and low quality interaction with the provider. These findings fit well within Andersen’s Behavioral Model of Health Service Use, as contraceptive access and care were influenced by systemic level factors within the health system, like wait times [23]. One in five mystery clients – all of whom presented as *new* family planning clients – were offered fewer than five minutes of counseling, suggesting that providers may not be allocating sufficient time for counseling new clients. Long wait times may contribute to these abbreviated counseling sessions, as providers rush to attend those still waiting. Our findings mirror those of Wagenaar et al.’s time-motion study of over 8000 primary care clinical interactions in Mozambique, which linked heavy patient caseload and insufficient allocation of health providers to long wait times, short consultations, and overall reductions in quality of care [8].

Across discussions with providers and clients, health workforce shortages were identified as a major contributor to wait times. Beyond insufficient human resource allocation, provider absenteeism and lateness may exacerbate shortages. Our concurrent measures of provider absenteeism demonstrate an absence rate of nearly 60% in participating facilities; among those providers who were present, nearly 20% were not working at the time of the visit [33]. Finding innovative ways to motivate providers to arrive on time and to attend to clients after lunch may help to address wait times even in the face of workforce shortages. At the same time, it is important to recognize that shortages in providers may contribute to burnout, which may lead to high rates of absenteeism, creating a vicious cycle. Thus, the policy implications of this study suggest the need to ameliorate sizeable workforce shortages among public facilities in Western Kenya, while also identifying those factors which may facilitate a high degree of provider absenteeism, both of which contribute to the heavy caseloads which appear to fuel long wait times.

We note two key limitations of this study. First, our focus group participants were primarily married women in their thirties. As such, their data may not capture the experiences of younger, unmarried women. Secondly, our mystery clients were experienced data collectors and therefore may not fully represent women from lower wealth or educational strata. Women with less education or financial resources may potentially find themselves passed over in the queue and may therefore experience a longer wait period compared to mystery clients. Our inability to represent their experiences via the mystery client methodology may have biased our estimated wait time in a downward direction.

Despite these limitations, this paper addresses methodological limitations of previous research, triangulating quantitative and qualitative approaches to strengthen the rigor and validity of findings. We intentionally braided prospective collection of quantitative data on prevalence and duration of waits with retrospective collection of qualitative data on the meaning providers, clients, and key informants made of these waits to develop a thorough and complex picture of wait times for family planning services in Western Kenya. To the best of our knowledge, our study is the first to integrate the perspectives of family planning clients, providers, and government officials in Western Kenya, providing a nuanced and robust description of the phenomenon of long wait times.

## Conclusions

Family planning is critical to population health and wellbeing, but many people seeking family planning face facility-level barriers, including long wait times to access services. Though wait times are a long-standing challenge facing quality family planning provision, they are not intractable. Addressing the upstream human resource challenges raised by participants is one key strategy for enhancing service delivery and user satisfaction, as well as supporting reproductive autonomy.

## Paper context

Long wait times pose a barrier to high quality family planning care and client satisfaction, yet existing literature has methodological limitations. We employed a mixed-methods approach to describe prevalence, length, causes, and impacts of family planning wait times. We found that family planning clients and providers perceive long wait times to be pervasive and impede quality of care. Addressing workforce shortages could decrease wait times and promote reproductive autonomy among women in Western Kenya.
